# Bovine Parainfluenza Virus 3 and Bovine Respiratory Syncytial Virus: Dominant Viral Players in Bovine Respiratory Disease Complex among Serbian Cattle

**DOI:** 10.3390/ani14101458

**Published:** 2024-05-14

**Authors:** Vesna Milićević, Sofija Šolaja, Dimitrije Glišić, Milan Ninković, Bojan Milovanović, Milan Đorđević, Snežana Ristevski, Filip Spasojević, Miroljub Dačić

**Affiliations:** 1 Institute of Veterinary Medicine of Serbia, Janisa Janulisa 14, 11000 Belgrade, Serbia; sofija.solaja@nivs.rs (S.Š.); dimitrije.glisic@nivs.rs (D.G.); milan.ninkovic@nivs.rs (M.N.); bojan.milovanovic@nivs.rs (B.M.); 2 Veterinary Clinic “Mladenovac”, Kralja Petra I 347, 11400 Mladenovac, Serbia; djordjevicvet@gmail.com; 3 Delta Vet Med doo, Vladimira Popovića 8A, 11000 Belgrade, Serbia; snezana.ristevski@deltaagrar.rs; 4 Al Dahra Srbija doo, Gorskih Jasenova 4, 11000 Belgrade, Serbia; filip.spasojevic@aldahra.com; 5 Veterinary Specialized Institute “Jagodina”, Boška Jovića 6, 35000 Jagodina, Serbia; vsijdacic@gmail.com

**Keywords:** bovine respiratory disease complex, bovine viral diarrhoea virus, bovine herpesvirus-1, bovine respiratory syncytial virus, bovine parainfluenza virus 3, Influenza D virus, prevalence, Serbia

## Abstract

**Simple Summary:**

This study thoroughly investigated viral pathogens associated with bovine respiratory disease complex (BRDC) in Serbian cattle using serum and nasal swab samples. Conducted in 2024 across 65 randomly selected dairy farms in Serbia, excluding ones with vaccinated cattle, this study categorized the farms by their size: small, medium, and large. Serum samples from adult cattle were tested for antibodies against BVDV, BHV-1, BRSV, and BPIV3, while nasal swabs from respiratory-symptomatic animals were PCR-tested for viral genome detection. The results showed seropositivity for all four viruses on all of the farms, with BPIV3 being universally positive. Medium-sized and large farms exhibited higher levels of seropositivity for BRSV and BHV-1 compared to small farms (*p* < 0.05). Our true seroprevalence estimates were 84.29% for BRSV, 54.08% for BVDV, 90.61% for BHV-1, and 84.59% for BPIV3 at the animal level. A PCR analysis of the nasal swabs detected BRSV (20%), BHV-1 (1.7%), BVDV (8%), and BPIV3 (10.9%), with no Influenza D virus found. This study provides crucial insights into viral pathogen prevalence and circulation in Serbian cattle with BRDC, emphasizing the importance of surveillance and control measures to manage respiratory diseases in cattle populations.

**Abstract:**

Bovine respiratory disease complex, a complex respiratory ailment in cattle, results from a combination of viral and bacterial factors, compounded by environmental stressors such as overcrowding, transportation, and adverse weather conditions. Its impact extends beyond mere health concerns, posing significant economic threats to the cattle industry. This study presents an extensive investigation into viral pathogens associated with BRDC in Serbian cattle, utilizing serum samples and nasal swabs. A cross-sectional study was conducted in 2024 across 65 randomly selected dairy farms in Serbia, excluding farms with vaccinated cattle. The farms were categorized by their livestock count: small (≤50 animals), medium (51–200 animals), and large (>200 animals). Serum samples from adult cattle older than 24 months were tested for antibodies against BVDV, BHV-1, BRSV, and BPIV3. Nasal swab samples from the animals with respiratory signs were tested using PCR for viral genome detection. The results showed seropositivity for all four viruses across all of the farms, with BPIV3 exhibiting universal seropositivity. Medium-sized and large farms demonstrated higher levels of seropositivity for BRSV and BHV-1 compared to small farms (*p* < 0.05). Our true seroprevalence estimates at the animal level were 84.29% for BRSV, 54.08% for BVDV, 90.61% for BHV-1, and 84.59% for BPIV3. A PCR analysis of the nasal swabs revealed positive detections for BRSV (20%), BHV-1 (1.7%), BVDV (8%), and BPIV3 (10.9%). Influenza D virus was not found in any of the samples. This study provides critical insights into the prevalence and circulation of viral pathogens associated with BRDC in Serbian cattle, emphasizing the importance of surveillance and control measures to mitigate the impact of respiratory diseases in cattle populations.

## 1. Introduction

Bovine respiratory disease complex (BRDC) refers to a multifactorial respiratory condition that affects cattle. It is caused by a combination of viral and bacterial pathogens, as well as environmental stressors such as overcrowding, transportation, and adverse weather conditions [1]. BRDC is a significant concern in the cattle industry as it can lead to substantial economic losses due to decreased productivity, high treatment costs [2], and a mortality rate that can reach 70% [3]. For example, even with yearly preventive vaccination, BRDC still imposes an estimated annual cost of approximately GBP 80 million on the UK economy [4]. Bovine respiratory syncytial virus (BRSV), bovine parainfluenza virus 3 (BPIV3), bovine viral diarrhoea virus (BVDV), bovine herpesvirus 1 (BHV-1), bovine adenovirus (BadV), and bovine coronavirus (BoCV) are recognized as the primary causes of respiratory illness in cattle [5]. Influenza D virus (IDV) was first identified in 2011 [6] and has since been confirmed in North America, Europe, East Asia, and Australia [7]. Although it typically causes mild symptoms, recent metagenomic analyses have shown a positive association between IDV and BRDC [8]. Viral infections further exacerbate the conditions conducive to bacterial infections. The damage to the upper respiratory tract and impaired mucociliary clearance enhance bacterial adhesion to virus-infected cells, facilitating their growth and colony formation. This damage progresses to the tracheal mucosa epithelium, enabling bacteria to penetrate deeper into the respiratory tract. Viruses also hinder the function of macrophages and neutrophil leukocytes, which are crucial for host immune responses and phagocytosis [9]. BRDC affects animals with a range of symptoms lasting up to five days. They include fever, lethargy, anorexia, coughing, nasal and ocular discharges, and, in severe cases, strenuous breathing, while bacterial pathogens can trigger an acute phase response with systemic symptoms, like fever, loss of appetite, and respiratory issues. Neonatal calf diarrhoea, which can occur with or without fever, is also linked to BRD and may emerge after significant damage to the intestinal submucosa [10]. Though biosecurity and antibiotics are also pillars, the control of BRDC relies on vaccines [11]. However, despite advancements, current BRD vaccines show limited efficacy as indicated by the development of clinical disease even in vaccinated animals due to factors like improper administration and storage, as well as challenges in vaccinating young calves [4]. BRDC remains a persistent and widespread challenge globally, with significant prevalence observed across Europe. In Serbia, governmental initiatives targeting BRDC are notably absent, leaving vaccination against the disease optional. Moreover, the dearth of comprehensive data concerning the prevalence and ramifications of BRDC within the country further exacerbates the challenge of effectively managing this complex. Therefore, this study sought to address this critical gap by estimating the seroprevalence of the viral infections deemed most significant within the BRDC complex.

## 2. Materials and Methods

This study was carried out using serum samples from the state’s annual leukosis survey. Ethical approval or consent to participate was therefore not required.

### 2.1. Study Design and Sampling

**Figure 1:**
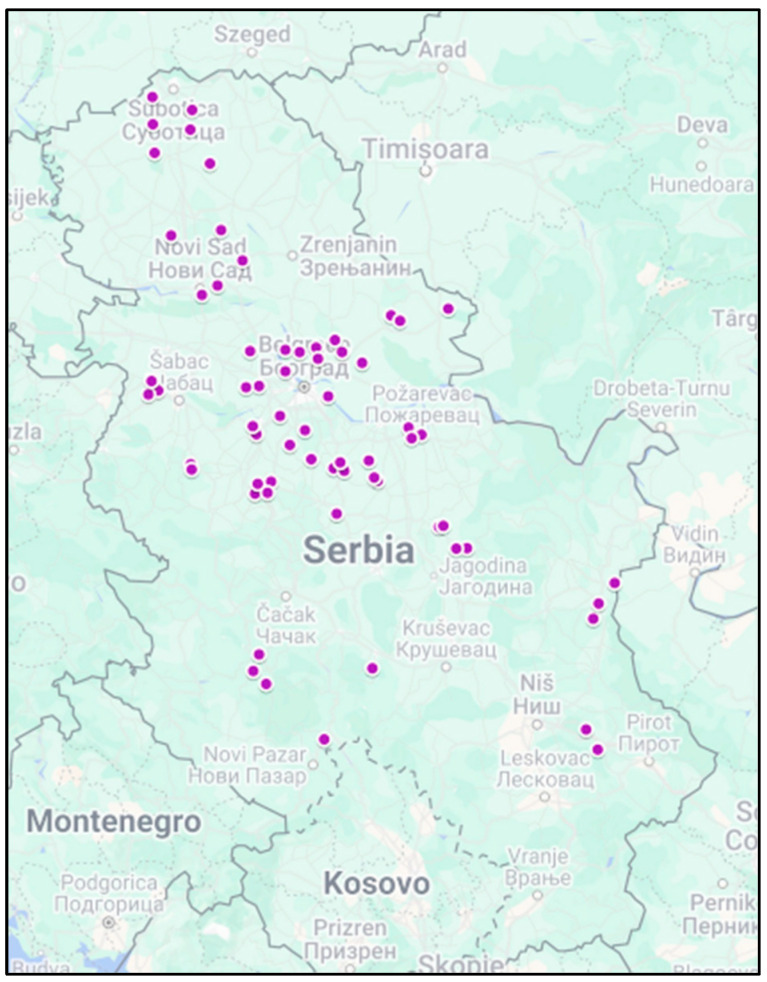
The purple dots represent the location of sampled cattle farms.

Serum samples were obtained from annual enzootic bovine leukosis surveys, which include testing of all adult cattle older than 24 months. This cross-sectional study was conducted during January and February 2024, encompassing 65 randomly selected dairy farms across Serbia where vaccination was not practised. The locations of the sampled farms are presented in Figure 1.

These farms were classified based on their livestock count: small farms with up to 50 animals (*n* = 50), medium-sized farms ranging from 51 to 200 animals (*n* = 10), and large farms housing over 200 animals (*n* = 5). The number of farms and individuals surveyed was determined based on a 2-stage sampling design. The herd-level sample size was calculated with an anticipated prevalence of 10%, a confidence level of 95%, and a precision of 0.05%, alongside data on the total number of cattle farms in Serbia obtained from the Statistical Office of the Republic of Serbia. The number of animals to be tested was determined to ensure 95% probability of detecting at least one positive animal if the herd is infected, considering the farm’s animal count, an assumed prevalence of 20%, a confidence level of 95%, and a precision of 0.05%. All cattle were subject to testing in small farms with up to ten animals. The nasal swabs were collected from animals exhibiting clinical signs of respiratory illness. Symptoms varied from mild to moderate, with no severe cases observed. In total, 175 nasal swabs were gathered and tested, along with 1000 serum samples comprising 400 from small farms, 300 from medium-sized ones, and 300 from large farms.

### 2.2. Antibody Detection Tests

Serum samples were analysed to identify antibodies against BVDV, BHV-1, BRSV, and BPIV3. Commercial ELISA kits were employed for assessing antibodies against BVDV (PrioCHECK™ Bovine BVDV Ab Plate Kit by Prionics, Schlieren, Switzerland), BHV-1 (ID Screen^®^ IBR gB Competition by IDvet, Grabels, France), and BRSV (INgezim BRSV Compac by Ingenasa, Madrid, Spain). Sample preparation and testing procedures were carried out in accordance with the guidelines provided by the respective test manufacturers. The interpretation of test results was conducted individually, adhering to the specified cut-off values provided by the test producers. Specific antibodies against BPIV3 were assessed by hemagglutination inhibition (HI) using 0.5% chicken red blood cells and 4 hemagglutination units of SF4 strain of BPIV3 genotype A (ATCC-VR 281, American Bioresearch, Gaithersburg, MD, USA) propagated in MDBK cell line (ATCC CCL-22). The serum samples were heat-inactivated prior testing at 37 °C for 30 min. The antibody titre was determined as the reciprocal of the highest serum dilution that completely inhibited hemagglutination. The initial serum dilution was set at 1:4, with the seropositivity cutoff level established at 4.

### 2.3. PCR

Nasal swab samples were subjected to PCR testing to detect the presence of BVDV, BHV-1, BRSV, IDV, and BPIV3 genomes. Viral nucleic acids were extracted from the swabs using the IndiSpin Pathogen kit (Indical, Laipzig, Germany) according to the manufacturer’s instructions, following immersion in 1ml of phosphate-buffered saline (PBS). Published primers for BVDV [12], BRSV [13], BHV-1 [14], IDV [15], and BPIV3 [16] were utilized for specific genome amplification (Supplementary material, Table S1). Real-time PCR was conducted using Luna^®^ Universal qPCR Master Mix (NEB, Ipswich, MA, USA) for BHV-1, while real-time RT-PCR was performed using Luna Universal Probe One-Step RT-qPCR Kit (NEB, USA) for BVDV, BRSV, and PI3. Samples with Ct values below 40 were considered positive. Gel-based RT-PCR was applied for IDV detection using OneStep RT-PCR kit (Qiagen, Hilden, Germany).

### 2.4. Statistical Analysis

Descriptive statistical methods were used for the analysis of results. The true seroprevalence was calculated using https://epitools.ausvet.com.au/trueprevalence (accessed on 13 March 2024), using imperfect test and 95% confidence level.

## 3. Results

None of the 65 farms tested seronegative for all four viruses (Supplementary material, Table S2). All of the farms tested seropositive for BPIV3. Additionally, all medium-sized and large farms were seropositive for BRSV and BHV-1. Conversely, significantly lower levels of seropositivity were observed for BVDV (*p* < 0.05) and BHV-1 (*p* < 0.05) in small farms (Table 1) compared to large and medium-size farms, respectively.

The true seroprevalence at the animal level of BRSV was estimated at 84.29% (81.81–86.5%, with a CI of 95%). The true seroprevalence of BVDV is estimated to be 54.08%, (50.92–57.21%, with a CI of 95%). Regarding BHV-1, the true seroprevalence was 90.61% (88.54–92.37%, with a CI of 95%). Lastly, for BPIV3, the true seroprevalence was 84.59% (82.14–86.78%, with a CI of 95%).

Of the 175 nasal swabs collected, 20% (*n* = 35) tested positive for BRSV, 1.7% (*n* = 3) for BHV-1, 8% (*n* = 14) for BVDV, and 10.9% (*n* = 19) for BPIV3 (Supplementary material, Table S2, Figures S1–S4). The farm detection rates varied across different pathogens: BVDV ranged from 11.11% to 26.67%, BHV-1 was detected in 10% of cases, BRSV showed a detection rate between 50% and 100%, and BPIV3 ranged from 3.33% to 20% in terms of detection frequency. BRSV circulation was verified in seven of the farms, comprising two large and five small ones. BHV-1 was confirmed at a single large farm. BVDV circulation was identified in three farms: two large and one medium-sized. BPIV3 was detected in ten farms, evenly split between five large and five medium-sized ones. Influenza D virus was not detected in any of the tested farms. A statistically significant (*p* < 0.05) frequency of BVDV and BPIV3 detection was observed for the large and medium-sized farms compared to that of the small ones.

The simultaneous circulation of BRSV and BPIV3 was detected at two large farms, which were BVDV- and BHV-1-seropositive. BVDV and BPIV3 were concurrent at one large and one medium farm. The simultaneous circulation of BVDV, BHV-1, and BPIV3 was confirmed at one large farm.

## 4. Discussion

This study represents the first comprehensive investigation of viral pathogens associated with BRDC in Serbian cattle. Unlike European countries where eradication or control programs may be in place, Serbian farms grapple with BRDC without such support. Serbia’s cattle structure is diverse, leading to varied outcomes, and the results are segmented based on farm size to reflect this diversity.

Although IDV has been reported to be circulating throughout Europe since 2012, the virus was not detected in any of the tested samples in this study. Previous reports have suggested that its seroprevalence could reach as high as 94.6% [17]. However, the positivity rate from nasal swab tests is usually less than 10% [17]. One limitation of this research was the lack of available serology tests that could accurately determine the prevalence of IDV infections in cattle in Serbia. Additionally, it is essential to consider the less-intensive cattle import market in Serbia compared to that in other EU countries, which may impact the spread of the virus and thus its absence in Serbia. It was shown that virus shedding occurs after the import of young cattle, which contributes to a broader diffusion in destination countries and facilitates viral spread through livestock trade [18]. Furthermore, although small farms in Serbia practice very low biosecurity measures, they tend to produce their own replacement cattle rather than purchasing them from larger farms, which can also limit the spread of IDV [19].

In contrast to IDV, BPIV3 emerged as the most prevalent respiratory viral pathogen in cattle in Serbia, irrespective of farm size. The seroprevalence of BPIV3 varies globally, reaching 100% at the herd level in places like Iran [20], and our study similarly found a herd seroprevalence of 100%. Furthermore, the BPIV3 genome was detected in 10.9% of nasal swabs in our study, aligning with previous research in Serbia, which reported a positivity rate of 6.7% using genome detection and virus isolation [21]. The average prevalence of BPIV3 in nasal swabs, as determined by PCR, was estimated at 7%, with age showing a significant influence but not farm type, which is consistent with our findings [22]. Considering the typical lack of control measures for BPIV3, even on large farms in Serbia, along with the virus’s characteristics, such as efficient horizontal transmission, propensity for sub-clinical infections, and potential for reintroduction into herds [23], these results were anticipated.

Like BPIV3, BRSV also exhibited a trend toward 100% seroprevalence at the herd level in medium-sized and large farms. However, seroprevalence was notably lower in small farms, with 50% showing seronegativity. Among all of the viruses circulating in cattle, BRSV exhibits the highest pathogenicity, manifesting with clinical signs ranging from mild to moderate or even subclinical [24]. Despite the potential for cattle on farms to remain free from clinical symptoms due to the subclinical nature of the disease [25], this study highlighted that small farms could maintain seronegativity under extensive conditions. Nonetheless, efficient inter-herd transmission contributed to a high true seroprevalence of 84.29%. These findings echo other studies’ findings, attributing the prevalence to year-round virus circulation [26] and repeated exposure leading to reinfections [27]. However, although the rate of positive nasal swabs from sick animals in our study was 20%, which was higher than those of other authors [28], it should be noted that viral RNA can be detected for up to 27 days [29], leading to an increased chance of detection.

While BPIV3 and BRSV are commonly reported in cattle, several countries or regions have achieved freedom from BoHV-1 through the implementation of eradication programs [30]. However, in Irish beef cattle, the herd-level seroprevalence to BHV-1 was as high as 90%, with a mean within-herd prevalence of 40% [31]. Consistent with findings in other studies [31,32], significant disparities in seroprevalence among small, medium-sized, and large farms were observed in this study. Reports from Estonia [32] indicated a substantial increase in herd prevalence with herd size, reaching 3.4% in the smallest category, consistent with previous studies from Serbia [33], while the mean within-herd prevalence was 37.8%, corresponding closely to our findings of 38.46%.

Moreover, seroprevalence tends to increase with age [33] due to latency and lifelong exposure to the virus. In this study, only animals aged 24 months and older were included in the seroprevalence estimation; thus, this association could not be confirmed. Contrary to our serological findings and owing to the nature of BHV-1, the virus itself was detected in only 1.7% of the nasal swabs from the sick animals. Similar observations were made in Slovenia, where the detection rate of BHV-1 was 0.75% [34]. However, considering age-related infection, the detection rate in young animals could be notably higher, as evidenced by findings from Poland, where the BHV-1 genome was detected in 36.5% of nasal swabs from young beef cattle [35].

While several European countries have successfully eradicated bovine viral diarrhoea virus (BVDV), leading to a reduced prevalence rate of 1.5% [36], this study reveals that BVDV seroprevalence remains high in Serbia and is particularly contingent on farm size. For herds with up to 50 animals, the seroprevalence was determined to be 16%, surpassing the prevalence observed in a study focused solely on backyard farms in the Belgrade area, where it was 3.8%, albeit with within-herd rates of up to 80% [19]. However, variations in prevalence are evident concerning region and farm management, but the within-herd prevalence usually approaches 100% [37]. Globally, the seropositivity rate stands at 42.77%, with dairy cattle demonstrating the highest prevalence at 48.68%. Notably, positive rates were more pronounced during summer (60.16%) and winter (63.44%), while cows exhibited a lower positivity rate compared to that of bulls, and calves showed a lower rate compared to that of adult cattle [36]. In comparison to antibodies, BVDV is considerably less detectable. In this study, only 8% of the nasal swabs from such animals contained BVDV, which is lower than the global average [36].

BPIV3 and BRSV were concurrently detected on two large farms, despite the presence of BVDV and BoHV-1, which are typically absent in order for BPIV3 and BRSV to become predominant viral pathogens, according to previous research [38]. A confirmation of the concurrent circulation of BVDV, BHV-1, and BPIV3 was obtained from a single large farm. The immunosuppression induced by BVDV infection facilitates other infections, resulting in a synergistic effect for numerous viral and bacterial pathogens responsible for respiratory illnesses.

Controlling the spread of BRDC in cattle populations is vital for their health and welfare. Key policies and practices include strict biosecurity measures to prevent infectious agent transmission, effective vaccination programs targeting common BRDC pathogens like BVDV, BHV-1, and BRSV, regular diagnostic testing to identify carriers, and implementing good management practices to reduce stress and minimize BRDC risk. These measures collectively aim to reduce disease prevalence and severity, ensuring healthier and more resilient cattle populations.

The study underscores the endemic nature and complex dynamics of viral pathogens associated with BRDC in Serbian cattle, influenced by factors such as farm size. Further research and targeted control measures are needed to mitigate the impact of these diseases on cattle health and productivity in Serbia.

## 5. Conclusions

This study emphasizes that viral pathogens linked to BRDC in Serbian cattle are not only prevalent but also exhibit intricate interactions, indicating an endemic nature. These pathogens, including BVDV, BHV-1, BRSV, and BPIV3, play a significant role in the occurrence and severity of BRDC. Importantly, the dynamics of these pathogens are influenced by various factors, with farm size being a notable factor. This underscores the importance of considering farm size as a factor in understanding and addressing BRDC in Serbian cattle herds. Further research is warranted to delve deeper into these complex dynamics, including investigating how farm management practices and environmental factors may also contribute to disease prevalence and transmission. Moreover, this study highlights the need for targeted control measures to effectively mitigate the impact of these diseases on cattle health and productivity in Serbia. This includes implementing vaccination programs, improving biosecurity measures, and enhancing surveillance efforts to monitor disease prevalence and detect outbreaks early. By better understanding the endemic nature and dynamics of these viral pathogens and implementing appropriate control strategies, it is possible to reduce the burden of BRDC on Serbian cattle herds, ultimately improving animal welfare and economic outcomes for cattle producers.

## Figures and Tables

**Table 1:** Seroprevalence of viral pathogens in Serbian cattle farms.

Farm Size	Number of Farms/ Number of Tested Animals	BRSV		BVDV		BHV-1		BPIV3	
		Seropositive Herds (number/%)	Seropositive Animals (number/%)	Seropositive Herds (number/%)	Seropositive Animals (number/%)	Seropositive Herds (number/%)	Seropositive Animals (number/%)	Seropositive Herds (number/%)	Seropositive Animals (number/%)
1–50 animals	50/400	40/80	331/82.75	8/16	197/49.25	10/20	325/81.25	50/100	379/94.75
51–200 animals	10/300	10/100	260/86.67	5/50	150/50	10/100	283/94.33	10/100	210/70
More than 200 animals	5/300	5/100	245/81.67	4/80	193/64.33	5/100	290/96.67	5/100	250/83.33
TOTAL	65/1000	55/84.61	836/83.6	17/26.15	540/54	25/38.46	898/89.8	65/100	839/83.9

## Data Availability

The data underlying this study are available upon request. Interested parties may contact the corresponding author for access to the dataset, subject to any confidentiality or data sharing agreements.

## Supplementary Materials

The following supporting information can be downloaded at: https://www.mdpi.com/article/10.3390/ani14101458/s1, Figure S1: qPCR_BHV-1; Figure S2: qRT-PCR_BPIV3; Figure S3: qRT-PCR_BVDV; Figure S4: qRT-PCR_BRSV; Table S1: Primers and probes sequences; Table S2: Row data results.
