# Supplementary figures and images for: Bovine Parainfluenza Virus 3 and Bovine Respiratory Syncytial Virus: Dominant Viral Players in Bovine Respiratory Disease Complex among Serbian Cattle
Source: Animals (Basel). 2024 May 14;14(10):1458. doi: 10.3390/ani14101458 (PMC11117219; doi:10.3390/ani14101458)

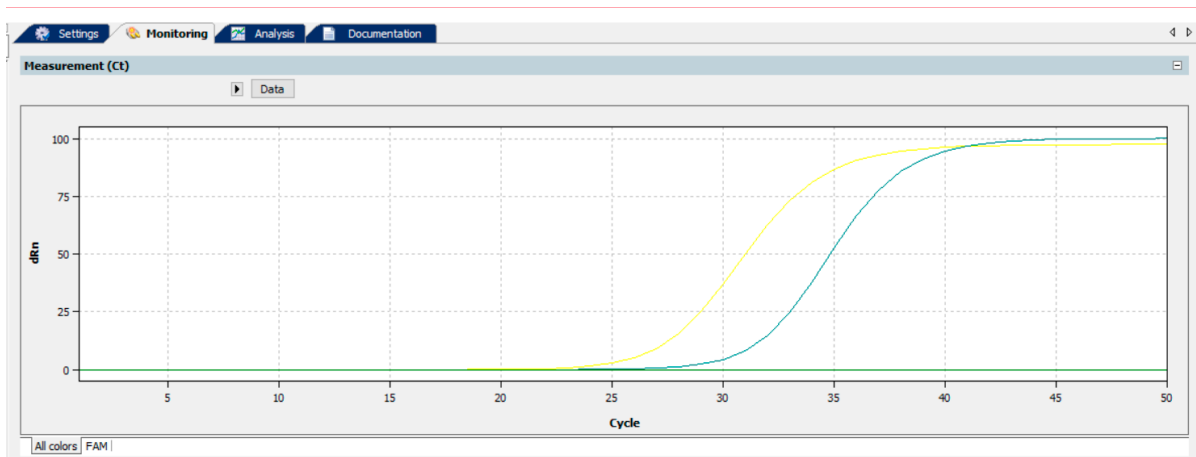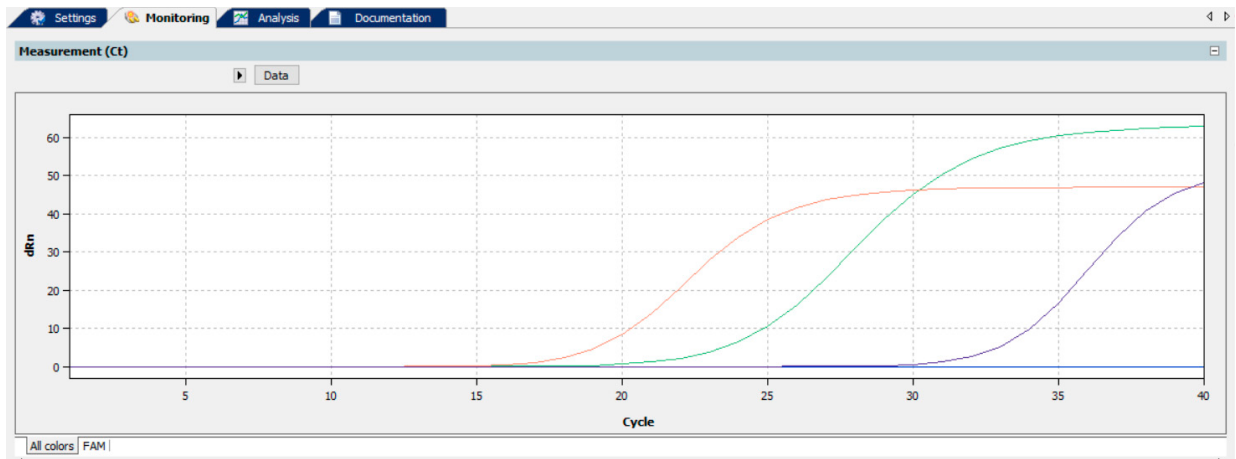

Supplement: Supplementary file 1 [file animals-14-01458-s001.zip › qPCR_BHV-1.pdf]

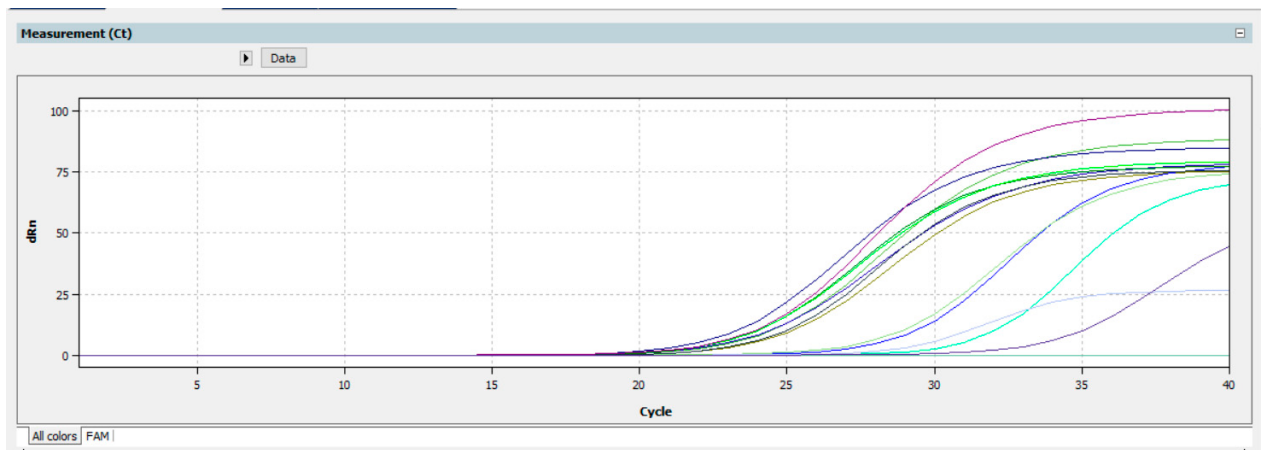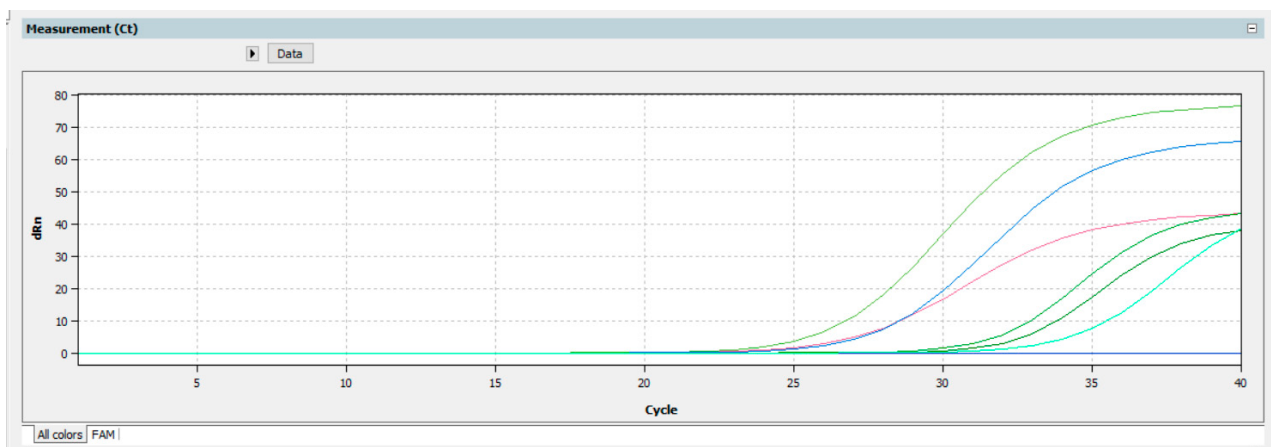

Supplement: Supplementary file 1 [file animals-14-01458-s001.zip › qRT-PCR_BPIV3.pdf]

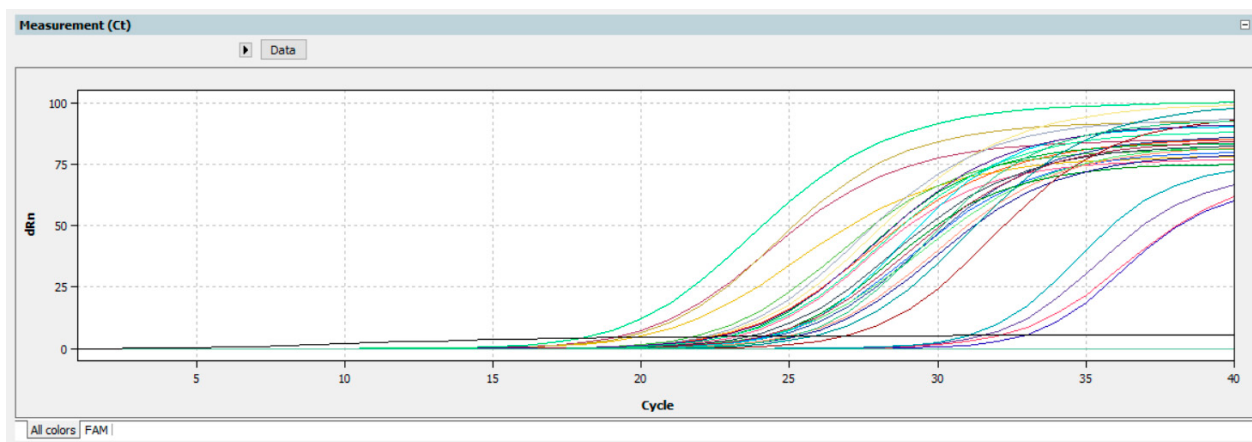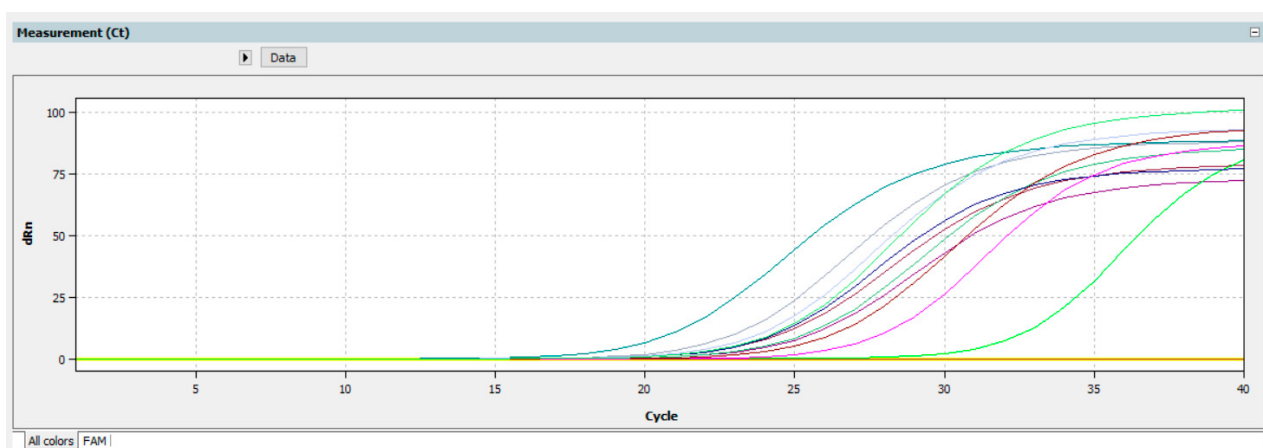

Supplement: Supplementary file 1 [file animals-14-01458-s001.zip › qRT-PCR_BRSV.pdf]

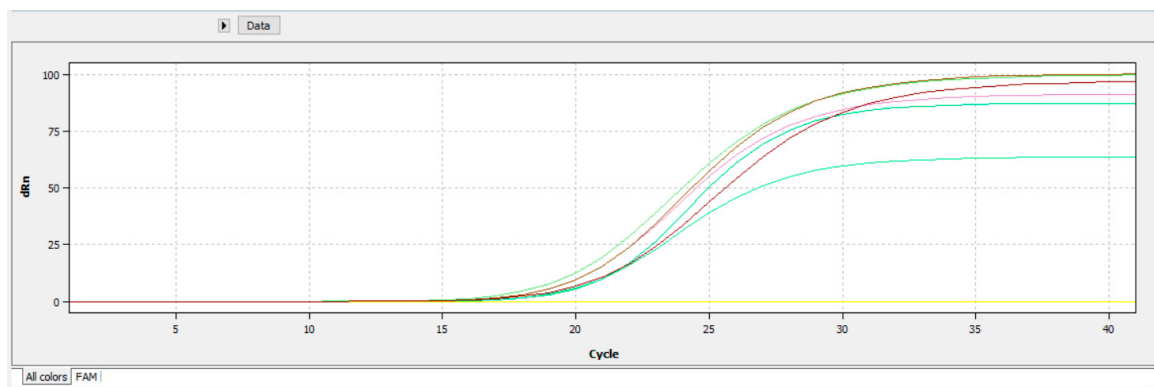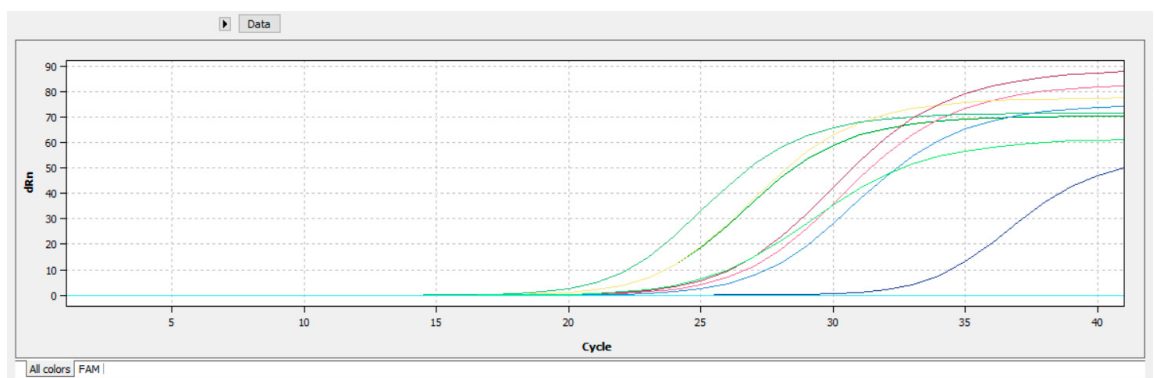

Supplement: Supplementary file 1 [file animals-14-01458-s001.zip › qRT-PCR_BVDV.pdf]
